# Inhibition of cytochrome P450 activities by juglone in human and rat liver microsomes: kinetic characterization and implications for natural product–drug interaction assessment

**DOI:** 10.3389/fphar.2026.1968844

**Published:** 2026-10-19

**Authors:** Luping Zhao, Yan Shen, Xin Xu, Lei Liu, Junze Huang, Chenmeizi Liang, Yuanjin Zhang, Xin Wang

**Affiliations:** 1 Shanghai Key Laboratory of Regulatory Biology, School of Life Sciences, East China Normal University, Shanghai, China; 2 Shanghai Institute of Wildlife Epidemics, East China Normal University, Shanghai, China

**Keywords:** cytochrome P450, Drug–drug interactions, enzyme kinetics, juglone, liver microsomes

## Abstract

**Introduction:**

Juglone is a redox-active constituent of walnut green husks, but its effects on drug-metabolizing cytochrome P450 (CYP) enzymes remain incompletely characterized.

**Methods:**

We evaluated juglone against six CYP activities in human and rat liver microsomes (HLMs and RLMs) using cocktail probe assays, time-dependent inhibition experiments, kinetic analyses, and molecular docking.

**Results:**

In HLMs, CYP2C9 was the most susceptible activity (IC_50_ = 8.12 μM; *K*_i_ = 4.97 μM) and showed competitive inhibition. CYP2E1 was the next most susceptible activity (IC_50_ = 12.67 μM; *K*_i_ = 19.59 μM; *αK*_i_ = 8.74 μM) and showed mixed inhibition. In RLMs, CYP1A2 (IC_50_ = 12.03 μM) and CYP2E1 (IC_50_ = 18.50 μM) met the prespecified criterion for further kinetic analysis. Subsequent analyses identified mixed inhibition of CYP1A2 (*K*_i_ = 12.31 μM) and competitive inhibition of CYP2E1 (Ki = 19.89 μM). No consistent progressive loss of activity was observed during preincubation for up to 20 min under the tested conditions. CYP2E1 showed numerically similar apparent *K*_i_ values but different fitted inhibition models in HLMs and RLMs. However, differing assay conditions precluded attributing these findings solely to species. Molecular docking provided qualitative structural insight into the potential interactions between juglone and CYP2C9.

**Conclusion:**

Juglone inhibited CYP activities *in vitro*, with CYP2C9 showing the greatest susceptibility among the six human CYP activities tested. These findings characterize biochemical inhibition and provide a basis for evaluating potential metabolic interactions involving juglone-containing products.

## Introduction

Juglone (5-hydroxy-1,4-naphthoquinone, Figure 1) is a redox-active naphthoquinone found in *Juglans* species and enriched in Pericarpium Juglandis, the green walnut husk known in Chinese medicine as Qinglongyi (Liu et al., 2022). Juglone and its derivatives have been investigated for antimicrobial, anti-inflammatory, and anticancer activities. Antimicrobial studies have reported inhibition of bacterial growth and quorum-sensing programs (Majdi et al., 2023; Ma et al., 2024). Inflammation models have linked juglone to reduced inflammasome-associated signaling and oxidative stress (Chen et al., 2025; Xu et al., 2025). In tumor models, juglone has altered cancer-cell metabolism and survival pathways (Tang et al., 2022; Hu et al., 2023; Luo et al., 2024; Wang et al., 2025). Juglone has also served as a natural-product scaffold for the design of pharmacologically active derivatives (Atanasov et al., 2021; Juang et al., 2024; Zhang et al., 2025). The quinone moiety can undergo redox cycling and electrophilic reactions with cellular nucleophiles, processes that may contribute to both biological activity and toxicity (Dos et al., 2021; Wang et al., 2021; Akutsu et al., 2023). The occurrence of juglone in medicinal plant material, together with its pharmacological activities and use as a scaffold for bioactive derivatives, provides a rationale for evaluating its effects on drug-metabolizing enzymes. In particular, defining which CYP activities are susceptible to juglone and characterizing the inhibition would help prioritize potential metabolic interactions for further investigation.

**FIGURE 1:**
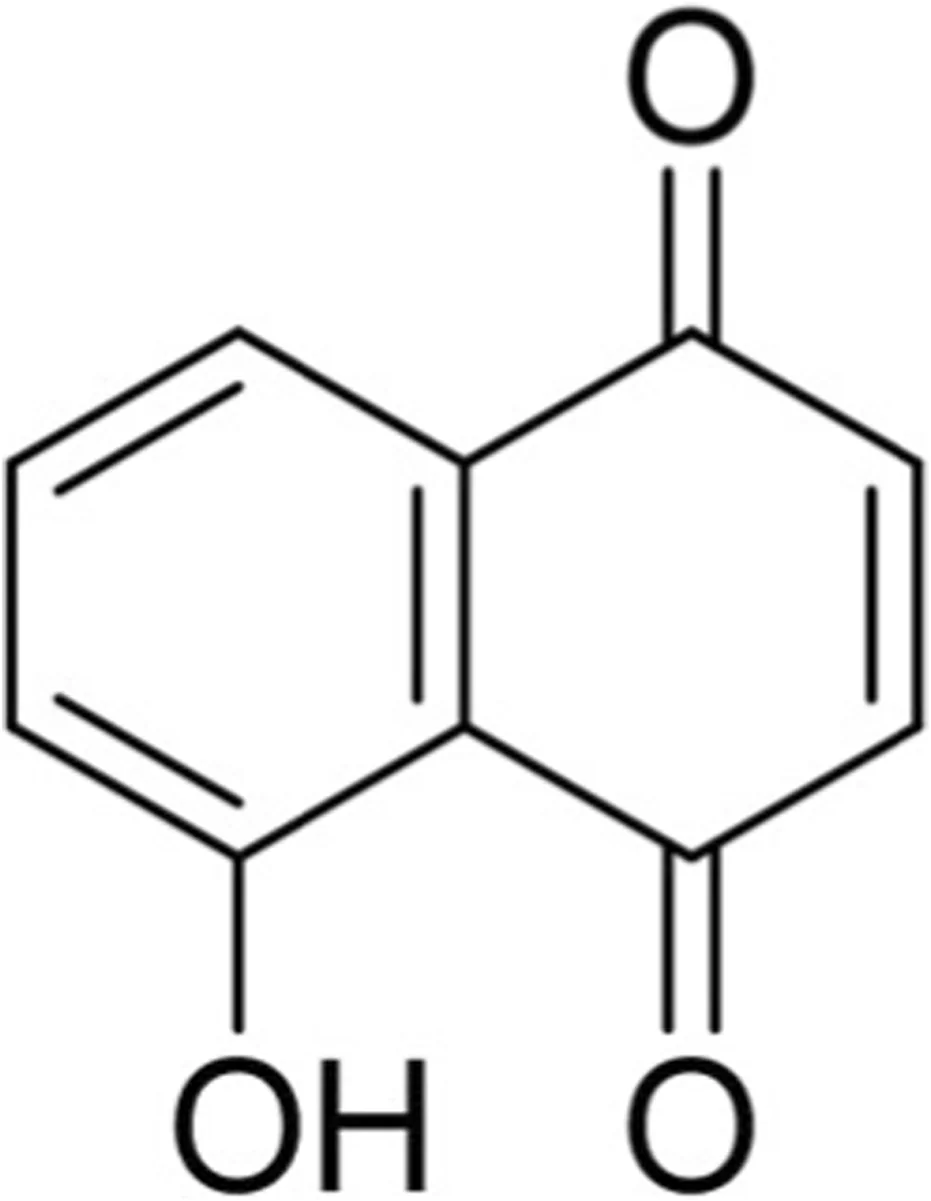
Chemical structure of juglone (5-hydroxy-1,4-naphthoquinone).

The cytochrome P450 (CYP) superfamily is a major enzymatic system responsible for the phase I oxidative metabolism of numerous xenobiotics, including clinically used drugs, as well as endogenous compounds (Zanger and Schwab, 2013; Almazroo et al., 2017; Hakkola et al., 2020; Guengerich, 2024). Among the major human drug-metabolizing isoforms, CYP1A2, CYP2B6, CYP2C9, CYP2D6, CYP2E1, and CYP3A4 contribute substantially to drug biotransformation and are routinely included in in vitro panels for evaluating CYP inhibition (He et al., 2023; U.S. Food and Drug Administration, 2023). Inhibition of these enzymes by a co-administered xenobiotic may decrease the metabolic clearance of substrate drugs and consequently increase their systemic exposure. The clinical relevance of such inhibition depends on several factors, including the unbound inhibitor concentration at the enzyme site, the contribution of the affected CYP isoform to the overall clearance of the substrate drug, and the drug’s therapeutic window (Yu et al., 2018; Hakkola et al., 2020; U.S. Food and Drug Administration, 2024). Herbal medicines, natural products, and dietary supplements are also potential sources of CYP inhibition, with evidence ranging from biochemical assays to clinical pharmacokinetic studies (Fasinu et al., 2012; Asher et al., 2017; Zhang et al., 2022; Oyanna and Clarke, 2024). Accordingly, microsomal IC_50_ and *K*_i_ measurements are useful for identifying isoform-selective biochemical inhibition and prioritizing potential interactions for further investigation, but these values alone cannot establish the occurrence or clinical significance of a drug–drug interaction (Wienkers and Heath, 2005; Rostami-Hodjegan and Tucker, 2007; Chen et al., 2020; Liu et al., 2023).

Previous studies from our laboratory characterized the inhibitory effects of the related naphthoquinones plumbagin, shikonin, and deoxyshikonin on CYP activities in human and rat liver microsomes (HLMs and RLMs), demonstrating inhibition across the six CYP activities examined in HLMs (Chen et al., 2016; Tang et al., 2017; Zhang et al., 2024). Given the CYP-inhibitory effects reported for related naphthoquinones, further investigation is warranted to determine whether juglone exhibits a similar inhibitory profile. Despite sharing a 1,4-naphthoquinone core, these compounds differ in their substitution patterns, and structural relatedness alone is insufficient to assign juglone’s isoform selectivity, inhibitory potency, or inhibition mode. The specific knowledge gap is therefore whether juglone exhibits similarly broad CYP inhibition or preferentially affects particular CYP activities, whether inhibition increases with preincubation time, and whether the susceptibility profile and inhibition kinetics differ between HLMs and RLMs. To address this gap, we evaluated juglone against six CYP activities in each microsomal system (CYP1A2, CYP2B6/2B1, CYP2C9/2C11, CYP2D6/2D1, CYP2E1, and CYP3A4/3A2) using a probe-substrate cocktail assay. Activities with IC_50_ values below 20 μM in the corresponding screen were selected for kinetic characterization and assessment of time-dependent inhibition. Molecular docking provided complementary qualitative structural context for the interaction with human CYP2C9. The contribution of this study is the integrated characterization of juglone’s CYP susceptibility profile, inhibition kinetics, and differences between human and rat microsomal systems, extending the evidence available for related naphthoquinones and identifying priorities for subsequent exposure-informed interaction assessment.

## Materials and methods

### Reagents and chemicals

Juglone (HPLC purity ≥98%) was obtained from Yuanye Bio-Technology (Shanghai, China). Phenacetin, tolbutamide, dextromethorphan, midazolam, chlorpropamide (internal standard), dexamethasone (internal standard), glucose 6-phosphate (G6P), glucose 6-phosphate dehydrogenase (G6PDH), and β-nicotinamide adenine dinucleotide phosphate (NADP^+^) were purchased from Sigma-Aldrich (St. Louis, MO, United States of America). Chlorzoxazone was purchased from Macklin Biochemical (Shanghai, China). Bupropion and dextrorphan were purchased from Topscience (Shanghai, China). 4-Acetamidophenol, hydroxybupropion, 4-hydroxytolbutamide, 6-hydroxychlorzoxazone, and 1-hydroxymidazolam were obtained from Toronto Research Chemicals (North York, Canada). Acetonitrile and ammonium formate (HPLC grade) were purchased from Fisher Chemicals (Leicester, UK). Formic acid was purchased from Adamas-beta (Shanghai, China). Pooled HLMs were purchased from BioIVT (INVITROCYP mixed-gender human liver microsomes; product no. X,008,070; lot: IVX) and stored at −80 °C until use. According to the supplier’s lot-specific characterization report, the preparation comprised microsomes from 150 donors, including 74 males and 76 females, aged 10 months to 85 years. The supplier-reported stock protein concentration was 22.8 mg/mL, and the total cytochrome P450 content was 0.365 nmol/mg protein. The supplier-reported activities for CYP1A2, CYP2B6, CYP2C9, CYP2D6, and CYP2E1 were 245, 75.5, 110, 57.4, and 492 pmol/min/mg protein, respectively. CYP3A4 activity, measured by midazolam 1-hydroxylation, was 330 pmol/min/mg protein. These activities were measured at substrate concentrations designated by the supplier as corresponding to apparent *K*_m_. Pooled RLMs were prepared previously in our laboratory from 10 male Sprague–Dawley rats (200–250 g; Shanghai SLAC Laboratory Animal, Shanghai, China). The present study used the same preparation batch as our previous study (Zhang et al., 2024). Liver tissue was rinsed with ice-cold saline and homogenized in Tris/KCl buffer (50 mM, pH 7.4). Microsomes were isolated by sequential centrifugation at 10,500 × *g* for 30 min and 105,000 × *g* for 60 min at 4 °C. The microsomal pellet was resuspended in Tris/KCl buffer, and protein concentrations were determined using a protein quantification kit. No separate in-house characterization of CYP activities was performed before the inhibition experiments. For both microsomal preparations, activities measured in vehicle-control incubations served as the reference for calculating relative CYP activity and inhibition. The supplier-reported HLM activities characterize the preparation under the supplier’s assay conditions and do not represent baseline activities measured under the present experimental conditions.

### Assessment of CYP inhibition by juglone in microsomal incubations

Microsomal incubations were conducted at 37 °C in a final volume of 100 μL. Pooled HLMs were used at a protein concentration of 0.5 mg/mL in potassium phosphate buffer (K_2_HPO_4_/KH_2_PO_4_, 100 mM, pH 7.4), whereas RLMs were used at 1 mg/mL in Tris–HCl buffer (50 mM, pH 7.4). The activities of CYP1A2, CYP2B6/2B1, CYP2C9/2C11, CYP2D6/2D1, CYP2E1, and CYP3A4/3A2 were measured using a CYP-selective probe-substrate cocktail assay. The probe substrates, screening concentrations, and monitored metabolites are summarized in Table 1. Probe selection was based on regulatory recommendations and established cocktail assays (Chen et al., 2016; Tang et al., 2017; U.S. Food and Drug Administration, 2023; Zhang et al., 2024). The final organic solvent concentration did not exceed 1% (v/v). Following a 5-min preincubation, reactions were initiated by adding an NADPH-generating system containing G6P (10 mM), G6PDH (0.4 U/mL), MgCl_2_ (5 mM), and NADP^+^ (1 mM), and incubation was continued for 20 min. Reactions were terminated by adding 300 μL of ice-cold acetonitrile containing chlorpropamide and dexamethasone as internal standards at final concentrations of 200 and 1,000 ng/mL, respectively. After vortex mixing, samples were centrifuged at 16,000 × g for 15 min at 4 °C. An 80-μL aliquot of the resulting supernatant was transferred to an autosampler vial, and 4 μL was injected into the LC-MS/MS system. For IC_50_ determination, juglone was tested at concentrations ranging from 1 to 100 μM under otherwise identical incubation conditions. No separate pre-experimental characterization of individual CYP catalytic activities was performed in our laboratory. Vehicle-control incubations provided the reference activities for calculating inhibition.

**TABLE 1:** Probe substrates and monitored CYP activities in the cocktail screening assay.

CYP activity	Probe substrate	Screening concentration (μM)	Monitored metabolite/activity
CYP1A2	Phenacetin	10	4-Acetamidophenol formation
CYP2B6/2B1	Bupropion	20	Hydroxybupropion formation
CYP2C9/2C11	Tolbutamide	20	4-Hydroxytolbutamide formation
CYP2D6/2D1	Dextromethorphan	5	Dextrorphan formation
CYP2E1	Chlorzoxazone	20	6-Hydroxychlorzoxazone formation
CYP3A4/3A2	Midazolam	10	1-Hydroxymidazolam formation

### Time-dependent CYP inhibition by juglone

Time-dependent inhibition was evaluated in a two-stage preincubation assay for CYP2C9 and CYP2E1 in HLMs and for CYP1A2 and CYP2E1 in RLMs (Obach et al., 2007; Faramarzi et al., 2025). HLMs (0.5 mg/mL protein) or RLMs (1 mg/mL protein) were incubated with juglone, an NADPH-generating system, and the appropriate buffer at 37 °C for 0, 5, 10, or 20 min. Juglone concentrations were selected to approximate the respective IC_25_ values in the secondary incubation after dilution. At each time point, an aliquot was diluted into a fresh 100-μL reaction mixture containing the relevant probe substrate and a newly prepared NADPH-generating system. The secondary incubation lasted 20 min. Reactions were quenched, processed, and quantified by LC-MS/MS as described above. No dedicated positive control for time-dependent CYP inhibition was included. Thus, the experiment was therefore interpreted as an initial screening assessment.

### Kinetic characterization of selected CYP activities

Activities with IC_50_ values below 20 μM in the corresponding microsomal screening assays were selected for kinetic characterization. Each kinetic experiment included four probe-substrate concentrations and four juglone treatment levels, with 0 μM serving as the vehicle control, as detailed in Table 2. Incubations were conducted for 20 min at 37 °C under the respective microsomal conditions described above.

**TABLE 2:** Substrate and juglone concentrations used in the selected enzyme kinetic experiments.

Microsomal system	CYP activity	Probe substrate	Substrate concentrations (μM)	Juglone concentrations (μM)
HLMs	CYP2C9	Tolbutamide	10, 20, 40, 80	0, 2, 5, 10
HLMs	CYP2E1	Chlorzoxazone	10, 20, 40, 80	0, 5, 10, 20
RLMs	CYP1A2	Phenacetin	5, 10, 20, 40	0, 2, 5, 10
RLMs	CYP2E1	Chlorzoxazone	10, 20, 40, 80	0, 5, 10, 20

### LC-MS/MS analysis

CYP probe substrates and their metabolites were quantified simultaneously using an LC-MS/MS method established previously, with minor modifications (Chen et al., 2016). Analyses were performed on an Agilent 1290 liquid chromatography system connected to an Agilent 6470 triple-quadrupole mass spectrometer equipped with an Agilent Jet Stream electrospray ionization (ESI) source. Agilent MassHunter software (version B.09.00) was used for instrument control, data acquisition, and data processing. Chromatographic separation was performed on a Phenomenex Kinetex XB-C18 column (100 × 3.00 mm, 2.6 μm) fitted with a C18 guard column (Torrance, CA, United States of America) and maintained at 30 °C. Mobile phase A was water containing 0.1% formic acid and 1 mM ammonium formate, and mobile phase B was acetonitrile containing 0.1% formic acid. Elution was performed at 0.3 mL/min using the following gradient: 10%–80% B from 0 to 2 min, 80%–90% B from 2 to 3 min, 90%–10% B from 3 to 4 min, and 10% B from 4 to 6 min. Detection used multiple reaction monitoring with positive/negative ion switching. The optimized MS/MS conditions and transitions were those reported previously. Calibration curves were constructed for all six metabolites using reference standards at known concentrations. For each metabolite, the metabolite-to-internal-standard peak area ratio (y) was regressed against the metabolite concentration (x, ng/mL) using linear regression. The calibration equations, concentration ranges, and coefficients of determination (R^2^) are provided in Supplementary Table S3. The six calibration curves yielded R^2^ values ranging from 0.990 to 0.999. Metabolite concentrations in incubation samples were calculated from the corresponding calibration equations using the measured metabolite-to-internal-standard peak area ratios.

### Molecular docking of juglone with CYP2C9 and CYP2E1

The crystal structures of human CYP2C9 (PDB ID: 5W0C) and CYP2E1 (PDB ID: 3E6I) were obtained from the RCSB Protein Data Bank (https://www.rcsb.org) (Burley et al., 2021). Both structures were prepared using the Protein Preparation Wizard in the Schrödinger Suite (Schrödinger, LLC, New York, NY, United States of America). For each protein, a receptor grid with a radius of 10.0 Å was centered on the co-crystallized ligand within the active site. The three-dimensional structure of juglone was obtained from the ChemicalBook database (https://www.chemicalbook.com) and prepared using LigPrep. Juglone was docked into both CYP binding pockets using Glide standard-precision mode (Friesner et al., 2004). The highest-ranked CYP2C9 pose was examined using an overall complex view, an enlarged active-site view, and a two-dimensional interaction map. For CYP2E1, the docking analysis was limited to the GlideScore, and no detailed pose or residue-level interaction analysis was performed.

### Data analysis

For each experimental condition, four reaction mixtures were separately prepared and incubated using the same pooled microsomal preparation. These constituted four technical incubation replicates (n = 4), not independent biological replicates. For the kinetic experiments, these replicates were obtained in four complete experimental runs, with one incubation per substrate–inhibitor concentration combination in each run. Incubation measurements are summarized as mean ± SD where indicated. The SD reflects variability among replicate incubations and does not estimate inter-individual biological variability. Fitted parameters and transformed data are presented as specified in the relevant tables and figure legends. Statistical analyses and curve fitting were performed in GraphPad Prism 10.1.2 (GraphPad Software Inc., CA, United States of America). Relative CYP activity (%) was calculated as 100 × *C*_juglone_/*C*_control_, where *C*_juglone_ and *C*_control_ represent metabolite concentrations in juglone-treated incubations and corresponding vehicle controls, respectively. IC_50_ values were estimated by variable-slope nonlinear regression of normalized activity against the logarithm of juglone concentration. For time-dependent inhibition assessment, the natural logarithm of residual enzyme activity was plotted against preincubation time. Kinetic data were fitted to the Michaelis–Menten model, and Lineweaver–Burk plots were used to visualize inhibition patterns. *K*_i_ values were estimated from secondary plots of the Lineweaver–Burk slopes (*K*_m_/*V*_max_) against juglone concentration; *αK*_i_ values were estimated from secondary plots of y-intercepts (1/*V*_max_) against juglone concentration. In addition, global nonlinear regression was performed, and the corrected Akaike information criterion (AICc) was used for model comparison to further assess the inhibition models and the consistency of the kinetic analysis.

## Results

### Inhibitory effects of juglone on CYP activities in HLMs and RLMs

Juglone was screened at 1–100 μM to determine IC_50_ values for six CYP activities in each microsomal system. In HLMs, CYP2C9 was the most strongly inhibited activity (IC_50_ = 8.12 μM), followed by CYP2E1 (12.67 μM) (Figure 2A; Table 3). CYP1A2 and CYP2B6 were less susceptible (49.94 and 54.29 μM, respectively), whereas CYP2D6 and CYP3A4 did not reach 50% inhibition within the tested range. In RLMs, CYP1A2 was the most strongly inhibited activity (IC_50_ = 12.03 μM), followed by CYP2E1 (18.50 μM), CYP2B1 (26.05 μM), CYP3A2 (26.19 μM), CYP2C11 (33.39 μM), and CYP2D1 (41.23 μM) (Figure 2B; Table 3). Kinetic analysis was therefore focused on CYP2C9 and CYP2E1 in HLMs and on CYP1A2 and CYP2E1 in RLMs, the activities with IC_50_ values below 20 μM in the respective screens.

**FIGURE 2:**
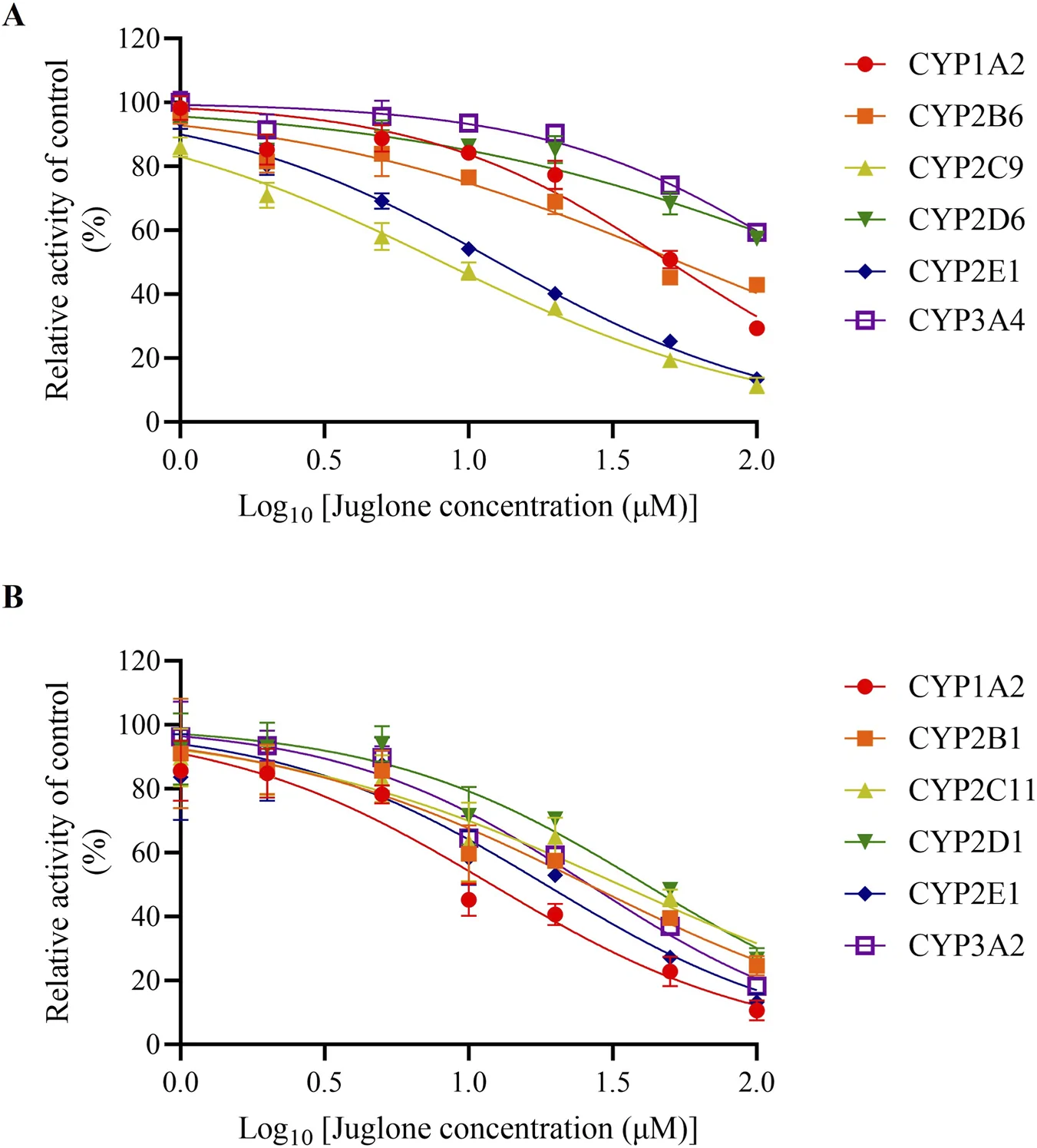
Concentration-dependent inhibition of CYP activities by juglone in human and rat liver microsomes. **(A)** CYP1A2, CYP2B6, CYP2C9, CYP2D6, CYP2E1, and CYP3A4 in HLMs. **(B)** CYP1A2, CYP2B1, CYP2C11, CYP2D1, CYP2E1, and CYP3A2 in RLMs. Activity is expressed relative to the solvent control. Juglone concentrations, expressed in μM, were log_10_-transformed before plotting. Data are presented as the mean ± SD of four technical replicate incubations. Concentration–response curves were fitted by nonlinear regression.

**TABLE 3:** IC_50_ screening results and selected kinetic parameters for juglone in human and rat liver microsomes.

Microsomal system	CYP activity	IC_50_ (μM)	*K*_i_ (μM) (95% CI)	*αK*_i_ (μM) (95% CI)	*α*	Inferred inhibition pattern
HLMs	CYP1A2	49.94	ND	ND	ND	ND
HLMs	CYP2B6	54.29	ND	ND	ND	ND
HLMs	CYP2C9	8.12	4.97 (1.971–11.73)	NA	NA	Competitive
HLMs	CYP2D6	>100	ND	ND	ND	ND
HLMs	CYP2E1	12.67	19.59 (13.54–29.67)	8.74 (6.006–12.46)	0.45	Mixed
HLMs	CYP3A4	>100	ND	ND	ND	ND
RLMs	CYP1A2	12.03	12.31 (9.318–16.87)	19.64 (15.12–26.80)	1.60	Mixed
RLMs	CYP2B1	26.05	ND	ND	ND	ND
RLMs	CYP2C11	33.39	ND	ND	ND	ND
RLMs	CYP2D1	41.23	ND	ND	ND	ND
RLMs	CYP2E1	18.50	19.89 (12.63–26.21)	NA	NA	Competitive
RLMs	CYP3A2	26.19	ND	ND	ND	ND

Kinetic analyses were focused on activities with IC_50_ values below 20 μM in the corresponding microsomal screen. *K*_i_ and *αK*_i_ are apparent estimates derived from secondary replots; values in parentheses are their corresponding 95% confidence intervals. *α* = *αK*_i_/*K*_i_. ND, not determined because kinetic analysis was not performed; NA, not applicable to the inferred inhibition pattern. The procedures used to obtain the confidence intervals are described in the Data analysis subsection and the to Supplementary Table S1.

### Evaluation of time-dependent CYP inhibition by juglone in HLMs and RLMs

Time-dependent inhibition was evaluated for CYP2C9 and CYP2E1 in HLMs and for CYP1A2 and CYP2E1 in RLMs. Preincubation with juglone for up to 20 min did not produce a consistent progressive decrease in residual activity in any selected assay (Figure 3). Therefore, no evidence of time-dependent CYP inactivation was observed under the tested conditions.

**FIGURE 3:**
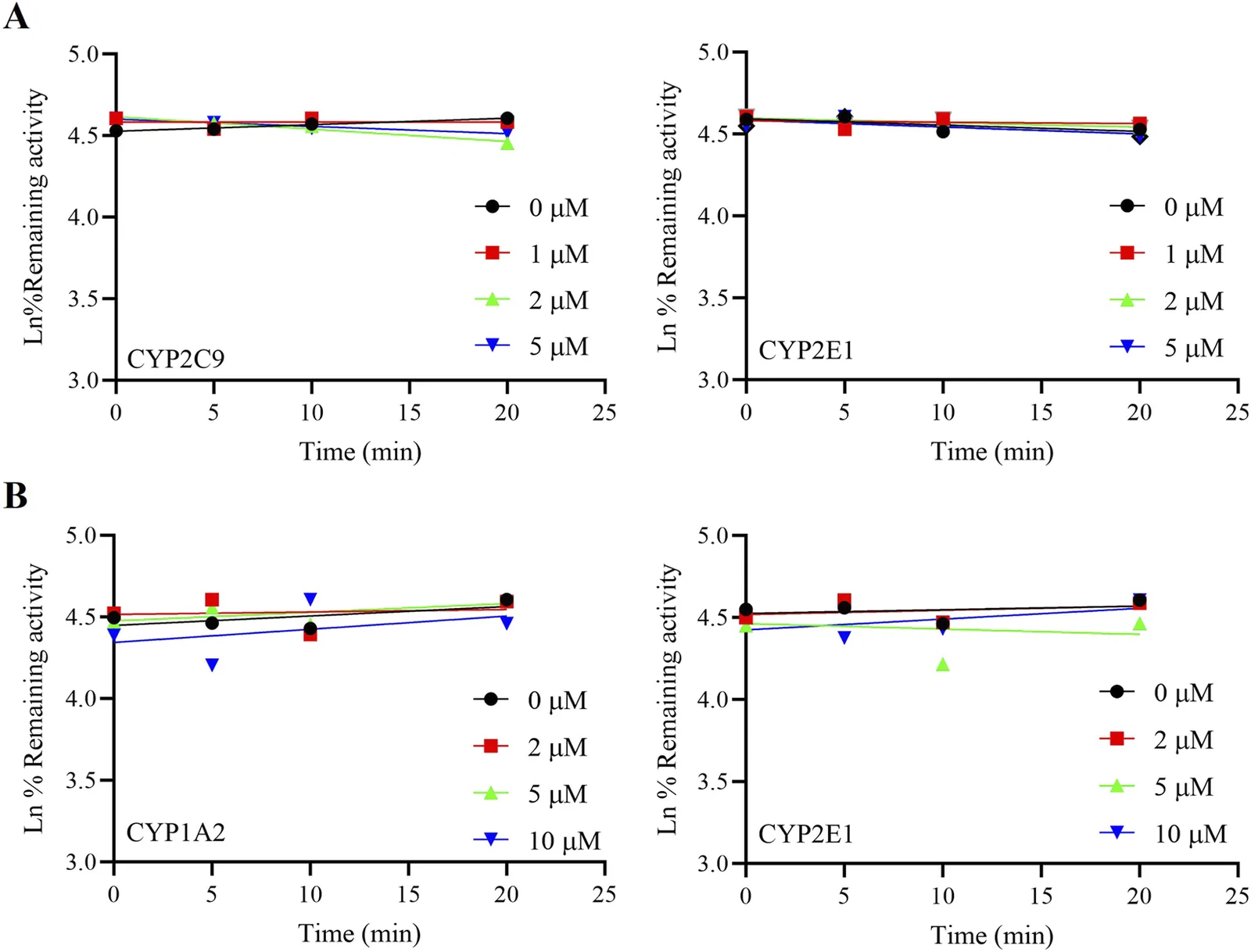
Time-dependent inhibition of selected CYP activities by juglone in liver microsomes. **(A)** CYP2C9 and CYP2E1 in HLMs. **(B)** CYP1A2 and CYP2E1 in RLMs. The natural logarithm of the percentage of remaining activity [ln (remaining activity, %)] was plotted against preincubation time in the presence of an NADPH-generating system. Data are presented as the mean ± SD of four technical replicates (n = 4) from the same pooled microsomal preparation.

### Kinetic characterization of CYP inhibition by juglone in HLMs and RLMs

Kinetic analyses used the concentration designs shown in Table 2. Lineweaver–Burk plots and secondary replots suggested competitive inhibition patterns for CYP2C9 in HLMs and CYP2E1 in RLMs. Mixed inhibition patterns were suggested for CYP2E1 in HLMs and CYP1A2 in RLMs. In HLMs, the apparent *K*_i_ was 4.97 μM for CYP2C9. For CYP2E1, the apparent *K*_i_ and *αK*_i_ values were 19.59 and 8.74 μM, respectively, with *α* = 0.45 (Figure 4). In RLMs, the apparent *K*_i_ and *αK*_i_ values for CYP1A2 were 12.31 and 19.64 μM, respectively, with *α* = 1.60. The apparent *K*_i_ for CYP2E1 was 19.89 μM (Figure 5). Parameter estimates are summarized in Table 3.

**FIGURE 4:**
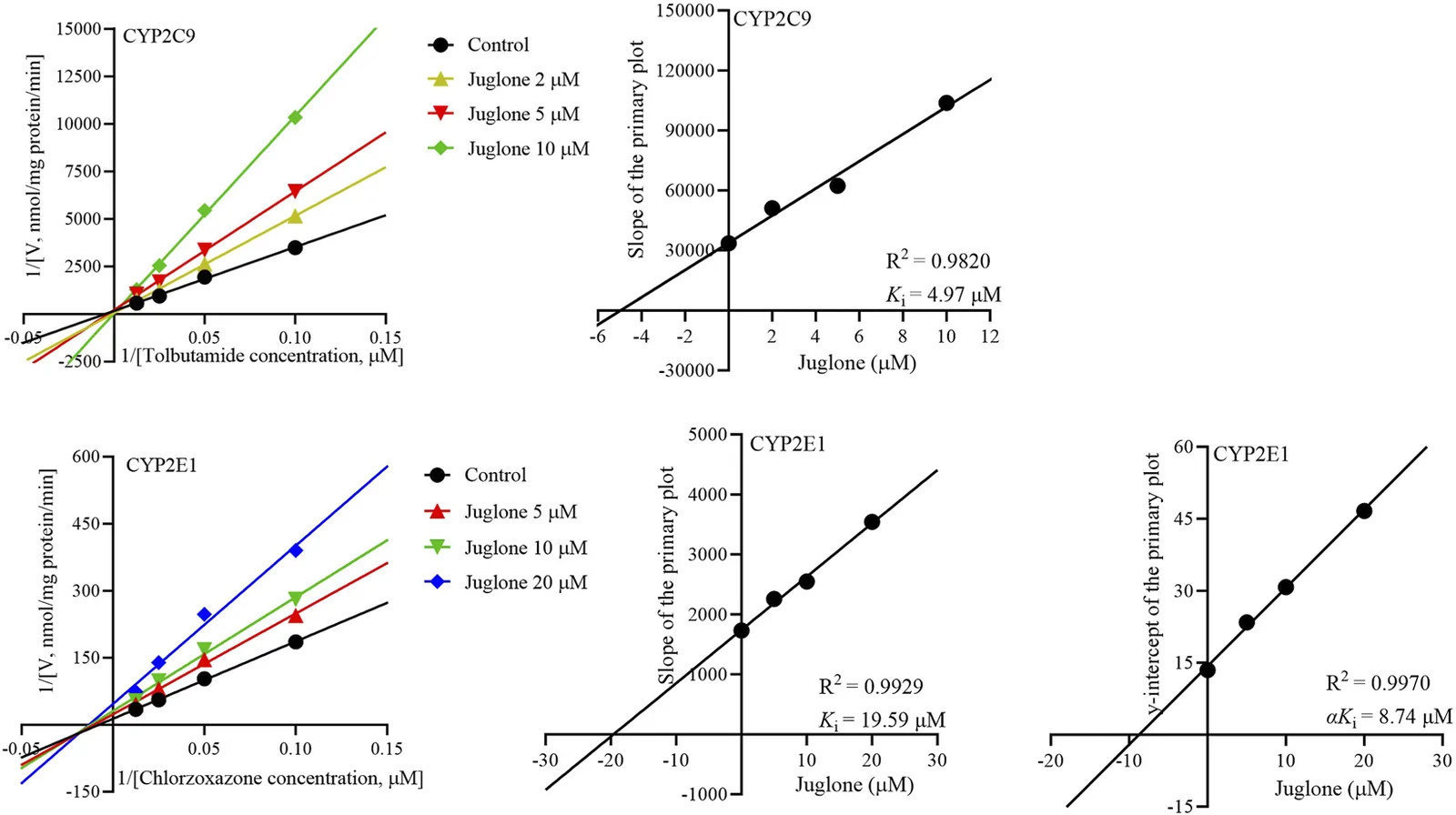
Kinetic characterization of CYP2C9 and CYP2E1 inhibition by juglone in HLMs. For each substrate–inhibitor concentration combination, four reaction mixtures were separately prepared and incubated using the same pooled HLM preparation (four technical incubation replicates). Points in the Lineweaver–Burk plots represent reciprocals of the mean reaction velocities from these four incubations; error bars are not shown. Points in the secondary replots represent slopes or y-intercepts estimated by linear regression of the corresponding Lineweaver–Burk plots. Secondary slope replots yielded apparent *K*_i_ values, and the y-intercept replot for CYP2E1 yielded an apparent *αK*_i_ value. Parameter estimates are summarized in Table 3. Complementary nonlinear fits are presented in Supplementary Figures S1, S2, with parameter and model comparisons in Supplementary Tables S1, S2.

**FIGURE 5:**
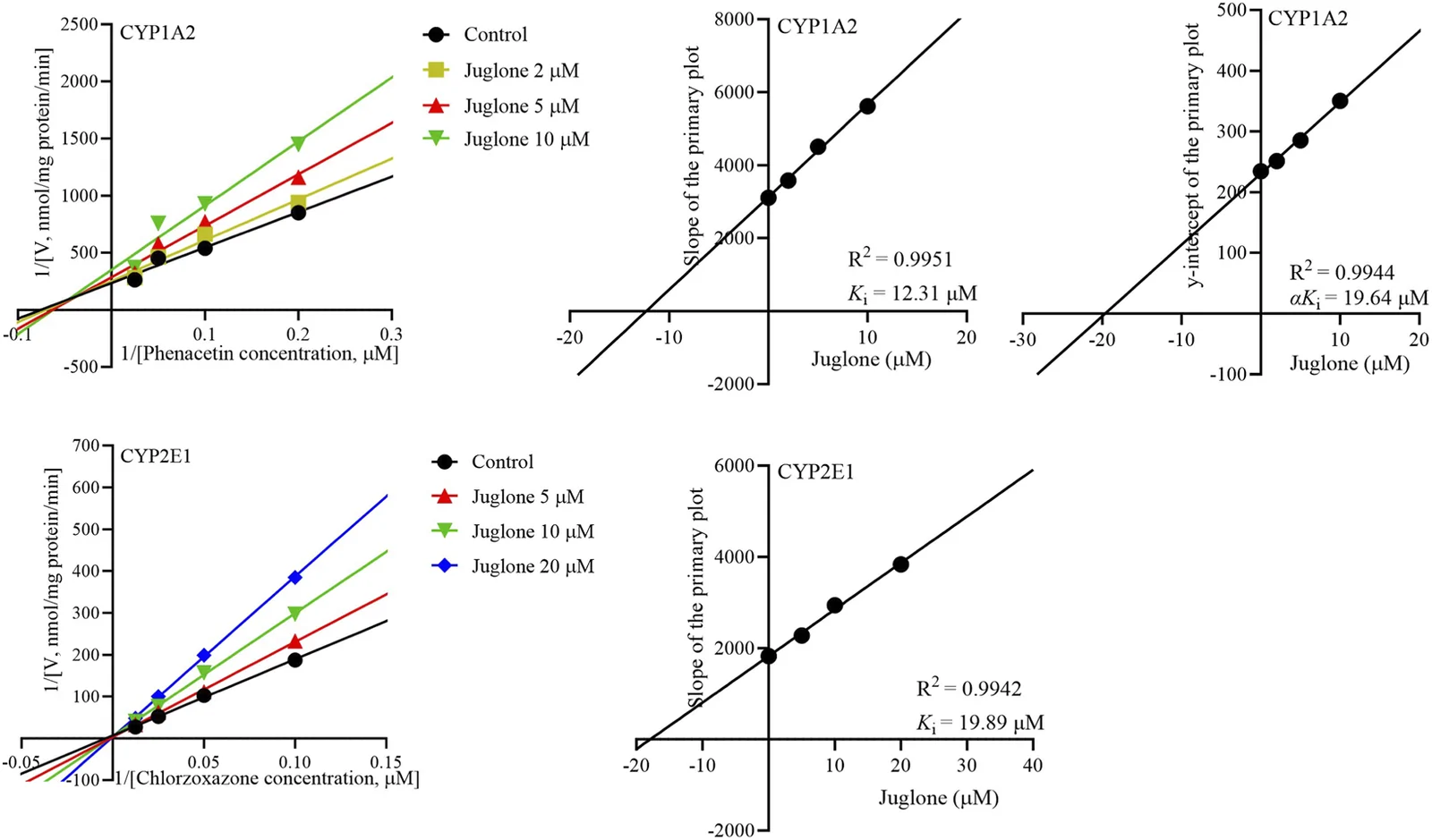
Kinetic characterization of CYP1A2 and CYP2E1 inhibition by juglone in RLMs. For each substrate–inhibitor concentration combination, four reaction mixtures were separately prepared and incubated using the same pooled RLM preparation (four technical incubation replicates). Points in the Lineweaver–Burk plots represent reciprocals of the mean reaction velocities from these four incubations; error bars are not shown. Points in the secondary replots represent slopes or y-intercepts estimated by linear regression of the corresponding Lineweaver–Burk plots. Secondary slope replots yielded apparent *K*_i_ values, and the y-intercept replot for CYP1A2 yielded an apparent *αK*_i_ value. Parameter estimates are summarized in Table 3. Complementary nonlinear fits are presented in Supplementary Figures S3, S4, with parameter and model comparisons in Supplementary Tables S1, S2.

### Molecular docking of juglone with human CYP2C9 and CYP2E1

Molecular docking yielded GlideScores of −6.758 kcal/mol for CYP2C9 and -7.502 kcal/mol for CYP2E1 (Table 4). For CYP2C9, the highest-ranked pose placed juglone close to the heme catalytic center (Figure 6). The model positioned the naphthoquinone ring near Phe114 and hydrophobic residues including Leu102, Ala103, Ala106, Leu208, Leu233, Val237, and Val292. A putative hydrogen bond involving Arg108 and a juglone carbonyl was also predicted. These predicted contacts have not been experimentally validated and are not used to assign inhibition type or rank inhibitory potency.

**TABLE 4:** Molecular docking scores of juglone toward human CYP2C9 and CYP2E1.

Human CYP isoform	GlideScore (kcal/mol)
CYP2C9	−6.758
CYP2E1	−7.502

**FIGURE 6:**
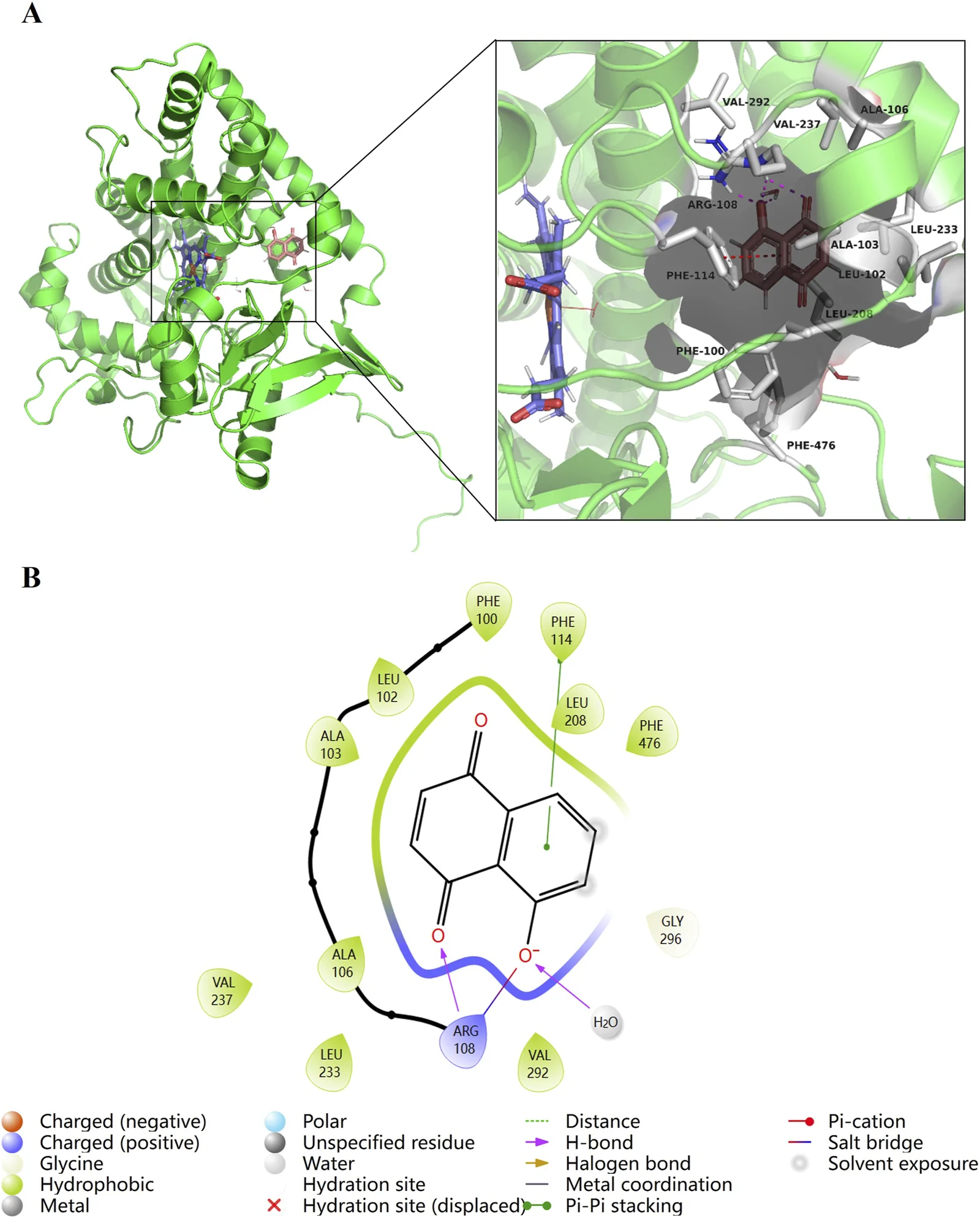
Qualitative molecular docking of juglone with human CYP2C9. **(A)** Overall and enlarged views of the predicted juglone pose in the CYP2C9 active pocket (PDB ID: 5W0C). **(B)** Two-dimensional interaction map of the predicted ligand–protein contacts. The pose and residue-level contacts are computational predictions and have not been experimentally validated; they do not establish inhibitory potency or the molecular mechanism underlying the observed kinetic pattern.

## Discussion

This study identifies CYP2C9 as the human CYP activity most susceptible to juglone among the six activities tested. Juglone also showed different inhibition profiles in HLMs and RLMs under their respective assay conditions. The combined screening and kinetic analyses define juglone’s CYP inhibition profile and identify CYP2C9 as a priority for investigating its metabolic interaction potential.

CYP2C9 showed the lowest IC_50_ among the tested human CYP activities and the lowest apparent *K*_i_ among those selected for kinetic analysis. The Lineweaver–Burk and secondary-replot patterns suggested competitive inhibition of CYP2C9-mediated tolbutamide metabolism. The lowest-AICc nonlinear model supported the same competitive interpretation, strengthening the kinetic evidence for this activity. This agreement concerns the inhibition pattern rather than the physical binding site of juglone. This finding is relevant because CYP2C9 contributes to the clearance of warfarin, sulfonylureas, phenytoin, and several nonsteroidal anti-inflammatory drugs (U.S. Food and Drug Administration, 2023; Guengerich, 2024). The microsomal IC_50_ and apparent *K*_i_ values quantify juglone-mediated CYP2C9 inhibition and provide an *in vitro* reference for subsequent exposure–inhibition comparisons. The CYP2C9 IC_50_ of 8.12 μM and apparent *K*_i_ of 4.97 μM correspond to approximately 1.41 and 0.866 μg/mL, respectively; these are nominal *in vitro* concentrations, not measured systemic exposures or clinical interaction thresholds.

Available pharmacokinetic evidence for juglone is primarily preclinical. A radiolabeled disposition study in male F344 rats estimated that approximately 40%–50% of an oral dose of 0.1–10 mg/kg was absorbed within 24 h and identified several urinary metabolites (Chen et al., 2005). These findings do not establish the plasma concentration or systemic bioavailability of unchanged juglone, because juglone-derived radioactivity may also represent metabolites. A preclinical formulation study reported a plasma half-life of approximately 2 h for non-liposomal juglone and an approximately 12-fold prolongation with liposomal delivery (Aithal et al., 2011), demonstrating formulation-dependent disposition rather than defining exposure during human use. An *in vitro* study reported greater than 99% binding to serum albumin (Zhao et al., 2017), highlighting the importance of distinguishing total from unbound concentrations. However, this finding does not by itself establish a human plasma unbound fraction suitable for quantitative prediction. Reliable human plasma *C*_max_ data for unchanged juglone under relevant use conditions were unavailable, preventing quantitative prediction of clinical interaction risk. Measuring unbound unchanged juglone is therefore a key next step in relating the observed CYP2C9 inhibition to human exposure.

CYP2E1 met the kinetic-selection criterion in both microsomal systems. Its apparent *K*_i_ values were numerically similar in HLMs and RLMs (19.59 and 19.89 μM, respectively), whereas reciprocal-plot analysis suggested mixed inhibition in HLMs and competitive inhibition in RLMs. However, the assays differed in microsomal protein concentration and buffer composition, factors that can influence apparent inhibitory potency and CYP catalytic activity (Tran et al., 2002; Tamemoto et al., 2025). The results identify CYP2E1 inhibition in both preparations and define the concentration range relevant to follow-up experiments. Similar apparent *K*_i_ values do not establish equivalent intrinsic susceptibility. Matched assay conditions and verified reaction linearity are needed to distinguish intrinsic species effects from assay-dependent differences.

In RLMs, CYP1A2 and CYP2E1 were the most susceptible activities, whereas CYP2C11 was less strongly inhibited under the tested conditions. CYP2C11 is not a one-to-one counterpart of human CYP2C9, and CYP abundance also differs between rat and human liver microsomes (Martignoni et al., 2006; Jiang et al., 2022). These considerations make the RLM profile useful for designing rat studies, while the HLM results provide the more direct basis for human CYP2C9 follow-up. The rat profile should not be treated as a quantitative surrogate for human CYP2C9 inhibition.

The preincubation experiments did not show a consistent progressive loss of the selected CYP activities over 0–20 min at the juglone concentrations tested. Preincubation followed by measurement of residual activity is an established approach for initial screening of CYP time-dependent inhibition (Obach et al., 2007; Wong et al., 2016). The limited preincubation period and concentration range, and the absence of a dedicated positive control for time-dependent inhibition, prevent definitive conclusions about reversibility or mechanism-based inactivation. Follow-up experiments should include an appropriate positive control and longer preincubation, with checks for enzyme stability and inhibitor depletion. Inactivation parameters (*k*_inact_ and *K*_I_), not determined here, would become relevant if reproducible time- and concentration-dependent inactivation is detected.

Previous studies from our laboratory reported inhibition of all six tested human CYP activities by plumbagin, shikonin, and deoxyshikonin (Chen et al., 2016; Tang et al., 2017; Zhang et al., 2024). In HLMs, juglone showed its strongest inhibitory effects against CYP2C9 and CYP2E1, with weaker inhibition of the other tested activities. The juglone profile extends the CYP inhibition evidence for this naphthoquinone series and identifies CYP2C9 and CYP2E1 as priorities for its further characterization. It also supports compound-specific assessment, because structural relatedness alone does not define a shared CYP inhibition profile.

Docking complemented the functional assays by identifying possible contacts between juglone and CYP2C9, including the predicted Arg108 interaction. The docking scores did not reproduce the experimental inhibition ranking. CYP2E1 yielded a more negative GlideScore than CYP2C9 (−7.502 versus −6.758 kcal/mol), whereas its apparent *K*_i_ in HLMs was higher (19.59 versus 4.97 μM). GlideScore is an empirical scoring function rather than an experimentally determined binding free energy, and its values cannot be directly translated into *K*_i_ estimates. The use of selected receptor conformations and approximate treatment of protein flexibility, solvation, and binding entropy limits quantitative interpretation of the scores (Friesner et al., 2004; Warren et al., 2006). The apparent *K*_i_ values describe inhibition of probe-substrate reactions in microsomes. The docking calculations did not model substrate competition or the enzyme–substrate–inhibitor states relevant to mixed inhibition. For human CYP2E1, the secondary-replot analysis yielded an apparent *αK*i of 8.74 μM under the mixed-inhibition interpretation. A single ligand–receptor score does not represent this kinetic parameter. Experimental inhibition data therefore determine potency rankings, while docking supplies structural hypotheses for testing. Since the docking grids were centered on the co-crystallized ligands within predefined active-site regions, the predicted CYP2C9 pose near the heme does not independently demonstrate competition at the substrate-binding site. The putative Arg108 hydrogen bond and other predicted contacts offer specific hypotheses for targeted binding or mutagenesis studies.

The microsomal experiments characterize inhibition at the enzyme-system level, without reproducing absorption, distribution, intact-cell transport, or systemic clearance (Li et al., 2021). The technical incubation replicates characterize variability within each pooled preparation but do not estimate inter-individual biological variability. Study-specific calibration curves for all six metabolites documented the relationships used for LC-MS/MS quantification (Supplementary Table S3). Comprehensive assay validation under the present conditions was not performed, leaving analytical bias and imprecision in the quantitative inhibition estimates incompletely characterized. The apparent *K*ᵢ and α*K*ᵢ estimates reported in Table 3 were derived from Lineweaver–Burk plots and secondary replots and are now accompanied by 95% confidence intervals. This graphical presentation illustrates inhibitor-dependent changes in slopes and intercepts and makes the derivation of the reported parameters transparent. These confidence intervals quantify uncertainty conditional on the secondary regressions but do not explicitly propagate uncertainty from the first-stage slope and intercept estimates.

Global nonlinear regression of untransformed reaction velocities provided a complementary assessment of the kinetic estimates and inhibition models (Supplementary Figures S1–S4; Supplementary Tables S1–S2). The nonlinear *K*_i_ estimates were broadly comparable in magnitude to the secondary-replot estimates. Model preference varied among CYP activities, but CYP2C9 retained the lowest estimated *K*_i_ among the human activities subjected to kinetic analysis. For human CYP2C9, the lowest-AICc competitive model agreed with the graphical interpretation and yielded a *K*_i_ of 5.11 μM, compared with 4.97 μM from secondary replots. For human CYP2E1, the mixed model was 2.27 AICc units above the lowest-AICc pure noncompetitive model, indicating a modest difference in relative model support. Mixed-model *αK*_i_ estimates were poorly constrained in some fits, particularly for the rat activities, limiting interpretation of those assignments. We retained the Lineweaver–Burk plots and secondary replots for their clear presentation of inhibitor-dependent changes in slopes and intercepts. The negative X-intercepts of the secondary replots explicitly connect the graphical analysis to the reported apparent inhibition constants. Together with the supplementary nonlinear fits, this presentation lets readers examine parameter estimates and alternative inhibition models. The constants remain method-dependent apparent estimates; numerical proximity does not establish equivalence between methods or remove the error weighting introduced by reciprocal transformation. Inhibition-model assignments describe kinetic behavior rather than establish molecular binding mechanisms.

The identification of CYP2C9 as the most susceptible human activity gives subsequent interaction studies a defined enzymatic focus. Translation requires measurements of juglone binding, metabolism, intracellular accumulation, and *in vivo* pharmacokinetics, which were not determined here. Recombinant human CYP2C9 experiments and measurements of plasma and microsomal binding would refine the inhibition estimates for exposure-based assessment. Together with suitable exposure data and statistically evaluated kinetic estimates, these measurements would support binding-adjusted predictions of interaction potential. Such predictions should account for the contribution of CYP2C9 to co-administered drug clearance (U.S. Food and Drug Administration, 2024). Metabolite identification and assessment of relevant metabolites for CYP inhibition would clarify contributions beyond the parent compound. *In vivo* pharmacokinetic studies should quantify unchanged juglone and relevant metabolites under defined dosing and formulation conditions. Overall, this study defines juglone’s CYP inhibition profile and prioritizes CYP2C9 for focused evaluation of its metabolic interaction potential.

## Conclusion

This study characterized the *in vitro* inhibition of six CYP activities by juglone in human and rat liver microsomes. CYP2C9 showed the greatest susceptibility among the six human CYP activities tested, with an IC_50_ of 8.12 μM and an apparent *K*ᵢ of 4.97 μM. Different inhibition profiles were observed in HLMs and RLMs, although differences in microsomal protein concentration and buffer composition prevent attributing these profiles solely to species. Assessment of clinical interaction potential requires human juglone exposure data and binding-adjusted inhibition estimates. Overall, these findings characterize juglone’s biochemical CYP inhibition profile and provide an experimental basis for further evaluating its potential metabolic interactions, with CYP2C9 identified as a priority for follow-up studies.

## Data Availability

The original contributions presented in the study are included in the article/Supplementary Material, further inquiries can be directed to the corresponding authors.
