# Supplementary material for: Inhibition of cytochrome P450 activities by juglone in human and rat liver microsomes: kinetic characterization and implications for natural product–drug interaction assessment
Source: Front Pharmacol. 2026 Oct 19;17:1968844. doi: 10.3389/fphar.2026.1968844 (PMC13724575; doi:10.3389/fphar.2026.1968844)
Supplement: Supplementary file 1 [file Table1.docx]

Supplementary Material

Figures S1–S4 present nonlinear kinetic fits. Tables S1 and S2 report inhibition constants and model comparisons. Table S3 provides LC-MS/MS calibration information.

**Supplementary figure legends**

**Figure S1.** Juglone inhibition of CYP2C9-mediated tolbutamide metabolism in human liver microsomes (HLMs). (A) Competitive, (B) mixed, (C) pure noncompetitive and (D) uncompetitive models. Juglone concentrations were 0 (control), 2, 5 and 10 μM. Symbols represent individual reaction-velocity measurements from four complete technical experimental runs, with four substrate concentrations at each of four inhibitor concentrations (64 observations per CYP activity). Lines represent run-specific fitted curves, not confidence bands. Global nonlinear regression used untransformed velocities and unweighted least squares in GraphPad Prism 10.1.2, with run-specific *V*_max_ values and shared *K*_m_ and inhibition constants. The same observations are shown in each panel. The competitive model had the lowest AICc. The mixed-model *αK*_i_ confidence interval had no finite upper limit. See Tables S1 and S2.

**Figure S2.** Juglone inhibition of CYP2E1-mediated chlorzoxazone metabolism in human liver microsomes (HLMs). (A) Competitive, (B) mixed, (C) pure noncompetitive and (D) uncompetitive models. Juglone concentrations were 0 (control), 5, 10 and 20 μM. Symbols represent individual reaction-velocity measurements from four complete technical experimental runs, with four substrate concentrations at each of four inhibitor concentrations (64 observations per CYP activity). Lines represent run-specific fitted curves, not confidence bands. Global nonlinear regression used untransformed velocities and unweighted least squares in GraphPad Prism 10.1.2, with run-specific *V*_max_ values and shared *K*_m_ and inhibition constants. The same observations are shown in each panel. The pure noncompetitive model had the lowest AICc; the mixed model had ΔAICc = 2.27 and no finite upper confidence limit for *αK*_i_. See Tables S1 and S2.

**Figure S3.** Juglone inhibition of CYP1A2-mediated phenacetin metabolism in rat liver microsomes (RLMs). (A) Competitive, (B) mixed, (C) pure noncompetitive and (D) uncompetitive models. Juglone concentrations were 0 (control), 2, 5 and 10 μM. Symbols represent individual reaction-velocity measurements from four complete technical experimental runs, with four substrate concentrations at each of four inhibitor concentrations (64 observations per CYP activity). Lines represent run-specific fitted curves, not confidence bands. Global nonlinear regression used untransformed velocities and unweighted least squares in GraphPad Prism 10.1.2, with run-specific *V*_max_ values and shared *K*_m_ and inhibition constants. The same observations are shown in each panel. The competitive model had the lowest AICc. The mixed-model fit approached the competitive-model boundary, and *αK*_i_ was not reliably identifiable. See Tables S1 and S2.

**Figure S4.** Juglone inhibition of CYP2E1-mediated chlorzoxazone metabolism in rat liver microsomes (RLMs). (A) Competitive, (B) mixed and (C) pure noncompetitive models. Juglone concentrations were 0 (control), 5, 10 and 20 μM. Symbols represent individual reaction-velocity measurements from four complete technical experimental runs, with four substrate concentrations at each of four inhibitor concentrations (64 observations per CYP activity). Lines represent run-specific fitted curves, not confidence bands. Global nonlinear regression used untransformed velocities and unweighted least squares in GraphPad Prism 10.1.2, with run-specific *V*_max_ values and shared *K*_m_ and inhibition constants. The same observations are shown in each panel. The competitive model had the lowest AICc among completed fits. The mixed-model fit approached the competitive-model boundary, and αKᵢ was not reliably identifiable. The uncompetitive model did not converge within 1,000 iterations using either of two starting-value sets. It was therefore omitted from the figure and excluded from AICc ranking. See Tables S1 and S2.

**Table S1.** Comparison of inhibition constants estimated by secondary replots and direct nonlinear regression.

| **CYP activity** | **Secondary-replot *K*_i_ (95% CI), μM** | **Secondary-replot *αK*_i_ (95% CI), μM** | **Lowest-AICc nonlinear model** | **Nonlinear *K*_i_**  **(95% CI), μM** |
| --- | --- | --- | --- | --- |
| CYP2C9 (HLMs) | 4.97  (1.971–11.73) | NA | Competitive | 5.11  (4.60–5.69) |
| CYP2E1 (HLMs) | 19.59  (13.54–29.67) | 8.74  (6.006–12.46) | Pure noncompetitive | 17.03  (15.80–18.41) |
| CYP1A2 (RLMs) | 12.31  (9.318–16.87) | 19.64  (15.12–26.80) | Competitive | 9.43  (6.98–13.14) |
| CYP2E1 (RLMs) | 19.89  (12.63–26.21) | NA | Competitive | 21.05  (19.39–22.89) |

Notes: Values in parentheses are 95% confidence intervals. Secondary-replot *K*_i_ and *αK*_i_ estimates were obtained from the negative X-intercepts of the slope-versus-inhibitor-concentration and Lineweaver–Burk y-intercept-versus-inhibitor-concentration replots, respectively. Their confidence limits were obtained by changing the signs of the corresponding X-intercept confidence limits and reversing their order. These intervals are conditional on the secondary regressions and do not explicitly propagate uncertainty from the first-stage slope and intercept estimates. Nonlinear estimates are from the lowest-AICc models among completed fits, with model-specific 95% profile-likelihood confidence intervals. The two types of confidence intervals are not interchangeable. Under the pure noncompetitive model, α*K*_i_ equals *K*_i_ and is not independently estimated. NA, not applicable.

**Table S2.** AICc comparison of candidate inhibition models fitted by global nonlinear regression.

**Human liver microsomes**

| **CYP activity** | **Inhibition model** | **AICc** | **ΔAICc** | **Fitting and parameter-identifiability notes** |
| --- | --- | --- | --- | --- |
| CYP2C9 (HLMs) | Competitive | −1260.16 | 0.00 | Lowest AICc |
| CYP2C9 (HLMs) | Mixed | −1257.74 | 2.42 | No finite upper 95% confidence limit for *αK*_i_ |
| CYP2C9 (HLMs) | Pure noncompetitive | −1256.50 | 3.66 | *αK*_i_ constrained to equal *K*_i_ |
| CYP2C9 (HLMs) | Uncompetitive | −1184.20 | 75.96 | *αK*_i_ confidence interval could not be determined |
| CYP2E1 (HLMs) | Pure noncompetitive | −914.48 | 0.00 | Lowest AICc; α*K*_i_ constrained to equal *K*_i_ |
| CYP2E1 (HLMs) | Mixed | −912.21 | 2.27 | No finite upper 95% confidence limit for *αK*_i_ |
| CYP2E1 (HLMs) | Competitive | −911.40 | 3.07 | Fit completed |
| CYP2E1 (HLMs) | Uncompetitive | −848.17 | 66.31 | *αK*_i_ confidence interval could not be determined |

Notes: Global nonlinear regression was performed in GraphPad Prism 10.1.2 using untransformed reaction velocities, expressed in nmol/mg protein/min. The nonlinear analysis included all 64 original measured velocities per CYP activity from four complete technical experimental runs. Run-specific *V*_max_ values were fitted, while *K*_m_ and inhibition constants were shared across runs. All candidate models for a given CYP activity were fitted to the same observations using unweighted least squares.

AICc denotes the small-sample-corrected Akaike information criterion. ΔAICc was calculated relative to the lowest AICc among completed fits for each CYP activity, using unrounded values. AICc comparisons apply within each CYP dataset, not across different CYP activities. The lowest AICc indicates relative preference among the evaluated models, not definitive identification of the inhibition mechanism.

**Table S2 continued.** AICc comparison of candidate inhibition models fitted by global nonlinear regression.

**Rat liver microsomes**

| **CYP activity** | **Inhibition model** | **AICc** | **ΔAICc** | **Fitting and parameter-identifiability notes** |
| --- | --- | --- | --- | --- |
| CYP1A2 (RLMs) | Competitive | −1043.52 | 0.00 | Lowest AICc |
| CYP1A2 (RLMs) | Mixed | −1040.90 | 2.62 | Competitive-boundary solution; *αK*_i_ not reliably identifiable |
| CYP1A2 (RLMs) | Pure noncompetitive | −1040.25 | 3.27 | *αK*_i_ constrained to equal *K*_i_ |
| CYP1A2 (RLMs) | Uncompetitive | −1028.65 | 14.87 | Fit completed |
| CYP2E1 (RLMs) | Competitive | −911.89 | 0.00 | Lowest AICc among completed fits |
| CYP2E1 (RLMs) | Pure noncompetitive | −910.73 | 1.15 | *αK*_i_ constrained to equal *K*_i_ |
| CYP2E1 (RLMs) | Mixed | −909.27 | 2.62 | Competitive-boundary solution; αKᵢ not reliably identifiable |
| CYP2E1 (RLMs) | Uncompetitive | Not available | Not ranked | Did not converge |

Notes: The analysis and AICc definitions are the same as those described for the HLM results in Table S2. For rat CYP1A2 and CYP2E1, mixed-model fits approached the competitive-model boundary, and *αK*_i_ could not be reliably identified. Their nominal AICc values are provided for transparency and should be interpreted cautiously because of boundary-related parameter non-identifiability. The rat CYP2E1 uncompetitive model did not converge within 1,000 iterations using either of two starting-value sets and was excluded from ranking.

**Table S3.** Calibration equations and ranges for the six metabolites measured by LC-MS/MS.

| **Metabolite** | **Calibration equation** | **R²** | **Calibration range (ng/mL)** |
| --- | --- | --- | --- |
| 4-acetamidophenol | y = 5.213x + 9.213 × 10^−4^ | 0.993 | 1–1000 |
| hydroxybupropion | y = 74.533x + 5.471 × 10^−2^ | 0.993 | 1–300 |
| 4-hydroxytolbutamide | y = 0.055x + 1.983 × 10^−5^ | 0.990 | 1–3000 |
| O-nordextromethorphan | y = 36.355x + 3.307 × 10^−2^ | 0.993 | 1–300 |
| 6-hydroxychlorzoxazone | y = 0.019x + 4.945 × 10^−5^ | 0.999 | 1–3000 |
| 1-hydroxymidazolam | y = 7.829x + 1.253 × 10^−2^ | 0.994 | 1–300 |

Notes: x represents the metabolite concentration (ng/mL), and y represents the metabolite-to-internal-standard peak-area ratio. R², coefficient of determination. The reported R² values range from 0.990 to 0.999. These calibration equations and ranges describe the calibration relationships used for metabolite quantification.
